# Three-dimensional ultrasound imaging of fetal brain fissures in the growth restricted fetus

**DOI:** 10.1371/journal.pone.0217538

**Published:** 2019-05-23

**Authors:** Sofie C. Husen, Irene V. Koning, Attie T. J. I. Go, Anne W. van Graafeiland, Sten P. Willemsen, Irene A. L. Groenenberg, Régine P. M. Steegers-Theunissen

**Affiliations:** 1 Department of Obstetrics and Gynaecology, Erasmus MC, University Medical Centre, Rotterdam, The Netherlands; 2 Department of Biostatistics, Erasmus MC, University Medical Centre, Rotterdam, The Netherlands; 3 Department of Paediatrics, Division of Neonatology, Erasmus MC Sophia Children’s Hospital, Rotterdam, The Netherlands; INSERM, FRANCE

## Abstract

**Objectives:**

To examine differences in growth trajectories of fetal brain fissures in the growth restricted fetus (FGR) compared to controls.

**Methods:**

We selected a subgroup of 227 women with a singleton pregnancy from the Rotterdam Periconceptional Cohort. Participants received three-dimensional ultrasound (3D-US) examinations of the fetal brain at 22, 26 and 32 weeks of gestational age (GA). The left and right Sylvian, insula and parieto-occipital fissures (POF) were measured in standardized planes. Linear mixed models with adjustment for potential confounders were applied to estimate differences between the trajectories of brain fissure depth measurements of FGR and controls.

**Results:**

22 FGR and 172 controls provided 31 and 504 3D-US respectively for longitudinal brain fissure depth measurements. Success rates for the Sylvian and insula depth measurements were over 80% and for POF over 62% at all GA. In FGR compared to controls, the trajectory of the right Sylvian fissure depth was significantly decreased (ß = -4.30, 95%CI = -8.03;-0.56, p = 0.024) while its growth rate was slightly increased (ß = 0.02, 95%CI = 0.00;0.04, p = 0.04), after adjustment for GA, head circumference, gender, educational level and parity.

**Conclusions:**

The small differences in brain fissure measurements between 22 and 32 weeks GA in FGR warrant further investigation in larger cohorts with postnatal follow-up.

## Introduction

Fetal growth restriction (FGR) affects 6–10% of pregnancies [1]. These fetuses are at risk for adverse pregnancy and neurodevelopmental outcomes, such as autism, attention deficit hyperactive disorder and schizophrenia [2, 3]. In FGR the ‘brain sparing effect’, referring to the cerebro-placental blood flow redistribution due to placental insufficiency measured with Doppler ultrasound, causes a change in oxygenation pattern in the fetus [4]. Since abnormal flow and oxygen patterns causing delayed cerebral development have been described in fetuses with congenital heart defects [5, 6], this change in blood flow could indicate an increased risk of developing brain abnormalities and subsequent neurodevelopmental disorders in FGR fetuses [4, 7, 8].

Cortical development of the brain takes place mainly during pregnancy. The process of development of new neurons and neuronal migration towards the outer brain surface is associated with cortical growth both in thickness and surface area [9]. This stress-induced development from a smooth to complex cerebral surface of sulci and gyri, called cortical folding, starts at around 18 weeks gestational age (GA) and is strongly correlated with GA [10].

The Sylvian fissure is the first fissure that can be seen on fetal MRI around 18 weeks of GA [11]. Other primary fissures, that appear on the brain surface, are the parieto-occipital fissure (POF), calcarine fissure and cingulate sulcus, which appear in the fetal brain between 18–24 weeks of gestation [11–14]. Development of the primary fissures start as a small dimple on the surface on the brain, after which the fissures become V-shaped and deepen [14]. At a later stage of fetal brain development ramifications of the primary fissures form the secondary and tertiary sulci [14].

The Sylvian fissure appears on the lateral convexities of the lateral hemispheres between the orbitofrontal lobes and the temporal lobes of which the tissue on the base of this fissure is called the Insula [15]. The POF is one of the prominent brain fissures which delineates the occipital lobe from the parietal lobe at the dorsomedial side of the brain [15].

Gender differences and left-right asymmetry in cortical folding have been demonstrated prenatally and have been classified as normal developmental phenomena [16, 17].

A different pattern of cortical folding may be related to abnormal cortical development of the brain [18, 19]. Therefore, prenatal analysis of these neurostructural changes of the cortex could be a potential predictor for (ab)normal neurodevelopment during fetal life and consequently neurodevelopmental functions in later life.

To evaluate cortical folding prenatally, several reliable imaging techniques including grading the tortuosity of brain fissures using two-dimensional (2D-US) and three-dimensional ultrasound (3D-US) and brain fissure depth measurements on MRI were investigated previously [10, 17, 20–28]. However grading the tortuosity of brain fissures using 2D-US is time-consuming. Evaluation of gyrification using MRI is potentially useful and can provide a ground truth for certain findings obtained by US, however MRI is time-consuming, rather expensive and not always readily available during pregnancy. Therefore, a more accessible technique and reliability of brain fissure depth measurements using 3D-US have been described previously by our research group [6].

Significantly deeper insular and left cingulate depths, decreased brain volume, a thinner insular cortex, smaller insula volume, reduction in cortical grey matter and altered cortical maturation have been described in FGR in previous studies [1, 2, 4]. Therefore, we aimed to investigate differences in brain fissure depth measurements between FGR and controls using the previously published technique [6]. In addition, Doppler indices of the major cerebral arteries were measured to relate brain fissure depth measurements to cerebro-placental flow indices.

## Materials and methods

### Study design and population

We selected the study population from the Rotterdam Periconceptional Cohort (Predict study), an ongoing prospective cohort study with follow-up until birth at the department of Obstetrics and Gynaecology at the Erasmus MC, University Medical Centre, Rotterdam, the Netherlands [29]. A subgroup of women with singleton pregnancies from the Predict study, willing to undergo longitudinal 3D-US examinations of the fetal brain in the second and third trimester of pregnancy, were enrolled prospectively between November 2013 and March 2015. Additionally, pregnancies with FGR fetuses were recruited during US examination between 18 and 32 weeks GA from the outpatient clinic. FGR was defined as an abdominal circumference or estimated fetal weight of less than the 5^th^ percentile according to the Hadlock 4 formula [30]. Exclusion criteria were withdrawal, intrauterine fetal death, termination of pregnancy, congenital malformations, and chromosomal abnormalities.

All participants and their partners signed written informed consent at enrolment on behalf of themselves and the unborn child. The study was approved by the regional Medical Ethical and Institutional Review Board of the Erasmus MC, University Medical Centre in Rotterdam (MEC 2004–227).

### Study parameters

Self-reported questionnaires were filled out and verified at enrolment, around 24 weeks GA and around delivery, containing data on maternal characteristics, pregnancy course and neonatal outcome. Pregnancy dating was based on crown-rump length (CRL), measured during a routine first trimester intake before 13 weeks GA [31]. IVF or ICSI pregnancies were dated from the date of the oocyte retrieval plus 14 days or date of the embryo transfer plus 17–18 days in cryopreserved transfers. Pregnancy outcome was validated with the report of the structural anomaly scan between 18 and 22 weeks and the medical delivery report.

### Ultrasound measurements

All participants volunteered for a longitudinal 3D-US evaluation, with a maximum of three examinations during second and third trimester at 22, 26 and 32 weeks GA. The 3D-US were performed on the Voluson E8 system (GE Medical Systems, Zipf, Austria) using a 1–7 MHz transabdominal transducer or a 6–12 MHz transvaginal transducer. Measurements of biparietal diameter (BPD), head circumference (HC), femur length (FL) and abdominal circumference (AC) were performed according to the ISUOG guidelines [32, 33] and an estimated fetal weight was calculated [30]. Doppler measurements of the umbilical artery (UA) and middle cerebral artery (MCA) were performed and used to calculate the cerebro-placental ratio (CPR).

**Fig 1:**
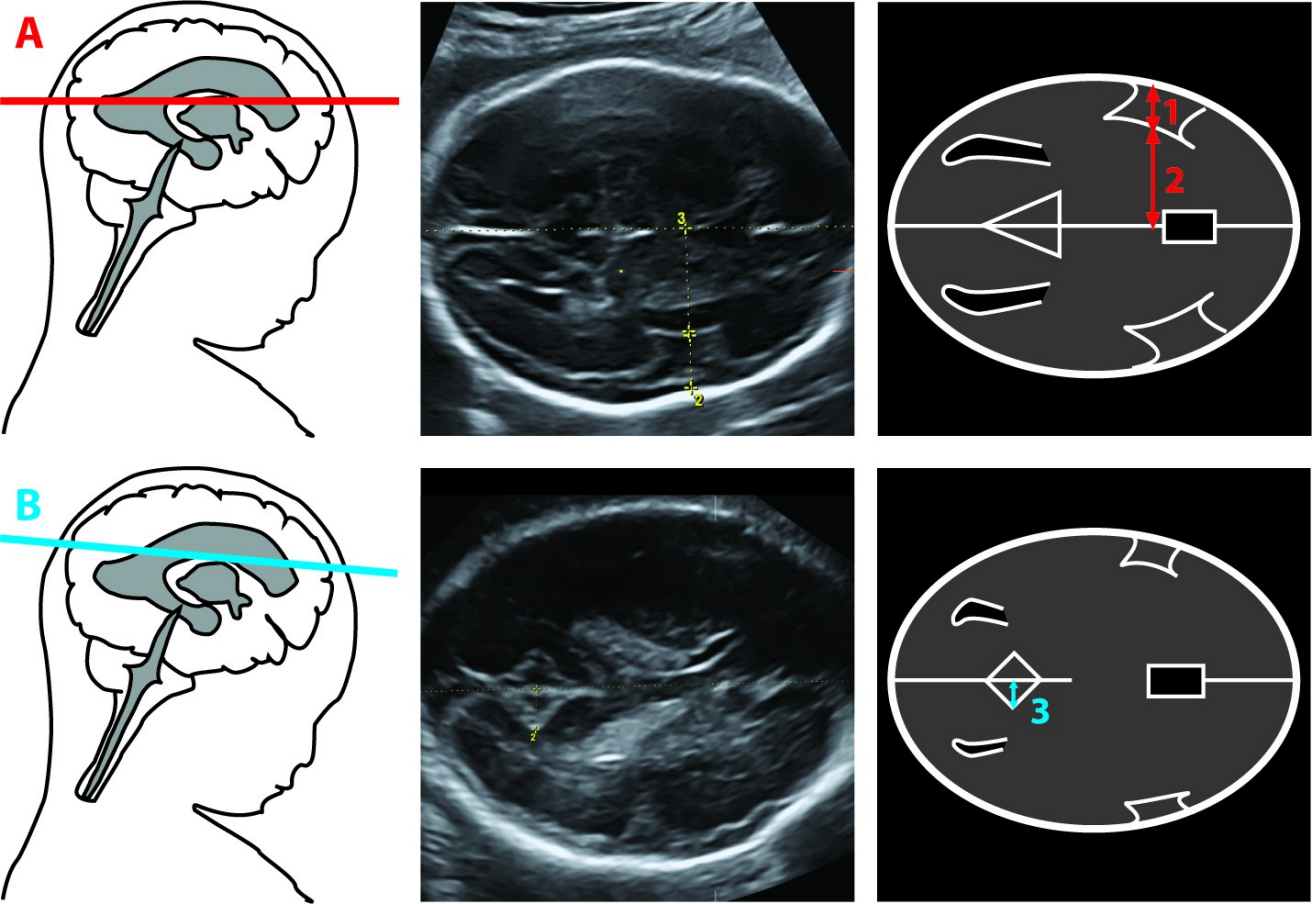
Brain fissure depth measurements using 3D-Ultrasound [6]. The measurement of the Insula depth (Fig 1A, measurement 2), the Sylvian fissure (Fig 1A, measurement 1) and The POF (Fig 1B, measurement 3).

The techniques and reliability of the brain fissure depth measurements have been described and published previously by our group [6]. The means of the Sylvian fissure and the POF measurement were significantly different between the two observers, although the mean percentage difference was only 6.2% and 7.5% respectively. The intra-observer analysis did not show significant differences between the measurements of the same observer. Intra- and inter-observer agreement of the sylvian fissure and insula were good, the agreement of the POF was acceptable [6]. In short a description of the used methods to perform the brain fissure depth measurements: Brain fissure depth measurements were performed offline using specialized 3D software (4D View, version 5.0, GE Medical Systems). All brain fissure measurements were performed perpendicular to the midline of the brain on which first a mid-baseline was drawn as a reference line to optimize precision. The insula and Sylvian fissure depth measurements were performed in the standard axial transventricular plane just above the trans-thalamic plane used for BPD and HC measurements, according to the ISUOG guidelines (Fig 1A) [20, 22, 24, 32, 33]. In an axial plane slightly above this transventricular plane the POF measurement was performed. This plane was slightly rotated along the z-axis, with the cavum septum pellucidum as a reference point, until the maximal depth of the POF was reached.(Fig 1B) [2, 23]. Reorientation of the 3D-US image according to a standard approach ensured differentiation between the left and right side [33]. A certified ultrasonographer carried out all ultrasounds (IVK) and all measurements were performed by one observer (AWG). The observer was blinded to the fetal group when identifying the fissures.

### Statistical analysis

Data analysis was performed using SPSS (SPSS release 21 for Windows, IBM, United States of America). Probability values below 0.05 were considered statistically significant. General characteristics of the FGR fetuses and controls were compared using Mann Whitney U-tests for continuous data, and Chi-square tests for categorical data.

To enhance precision and limit variance, all US measurements were repeated three times. We calculated the means for the statistical analyses and calculated success rates, medians and ranges for all brain fissure measurements per GA. The repeated brain fissure depth measurements at 22, 26 and 32 weeks GA were combined into growth trajectories to evaluate the process of cortical folding. Linear mixed models were applied to estimate the associations between FGR and the repeated brain fissure depth measurement. In the model we used a random intercept and a scaled identity matrix to fit the covariance structure. We studied the effect of polynomials to investigate the best model fit. Firstly, “Model 1” for all brain fissure depth measurements was estimated using the longitudinal brain fissure depth measurements as response and GA and square of GA as predictors. FGR and the interaction term of FGR with GA were used as independent predictors of the longitudinal brain fissure measurements. FGR represents the constant effect of FGR on the height of the growth trajectories. The interaction term of FGR*GA represents the effect of FGR on the slope of the trajectories, or in other words the growth rates of the brain fissures. Secondly, in the multivariate model (Model 2) designated confounders were entered simultaneously in the fully adjusted model. Potential confounders were selected from recent literature, i.e. HC and gender [22, 34] and from the list of general characteristics that were significantly different between FGR and controls. All confounders were included as covariates and used to investigate their independent associations with brain fissure measurements. In multivariate Model 3 we also adjusted for CPR to relate brain fissure depth measurements to the Doppler measurements and the brain sparing effect.

## Results

### Study population

227 singleton pregnancies were recruited from the Predict cohort. After exclusion according to the criteria described earlier, 194 patients were eligible for this study. Fetuses without ultrasound scans due to withdrawal (n = 6), termination of pregnancy (n = 2) or congenital anomalies (n = 25) were excluded. Table 1 depicts the baseline characteristics of the 194 pregnancies analysed in this study comprising FGR fetuses (n = 22) and controls (n = 172). Besides educational level and parity, there are no statistically significant differences in the baseline maternal characteristics between the FGR group and the control group. As expected, FGR fetuses were born with a shorter GA and lower birth weight. 22 FGR fetuses and 172 controls provided 31 and 504 3D-US respectively for brain fissure depth measurements.

**Table 1:** General characteristics of the study population and the subgroups FGR and controls.

	Total (n = 194)	FGR (n = 22)	Control (n = 172)	Missing	P-value
***Maternal characteristics***					
**Maternal age at enrolment, years, mean±SD**	32.0 ± 4.9	30.2 ± 5.7	32.3 ± 4.8	4	0.42
**Nulliparity, n(%)**	85 (45.0)	13 (68.4)	72 (42.4)	2	**0.03**
**Geographical origin, n (%)**					
Dutch	141 (75.0)	15 (79.0)	126 (75.0)	3	0.52
Other Western	11 (5.9)	0 (0)	11 (6.5)		
Non Western	36 (19.1)	4 (21.0)	32 (18.9)		
**Pre-pregnancy BMI, kg/m**^**2**^**, median (range)**	23.0 (15.2–43.4)	22.9 (17.6–43.4)	23.0 (15.2–39.7)	12	0.72
**Educational level, n (%)**					
low	25 (13.3)	4 (20.0)	21 (12.4)	3	**0.02**
middle	76 (40.4)	12 (63.2)	64 (37.9)		
high	87 (46.3)	3 (15.8)	84 (49.7)		
**Mode of conception, n(%)**					
Spontaneous	138 (71.9)	19 (90.5)	119 (69.6)	1	0.05
IVF/ICSI	54 (28.1)	2 (9.5)	52 (30.4)		
**Folic acid supplement use, n(%)**					
preconceptional initiation	128 (71.5)	10 (66.7)	118 (72.0)	8	0.80
postconceptional initiation	49 (27.4)	5 (33.3)	44 (28.0)		
**Periconceptional smoking, n(%)**	31 (16.6)	3 (15.8)	28 (16.7)	4	0.92
**Periconceptional alcohol use, n(%)**	50 (26.9)	4 (21.1)	46 (27.5)	5	0.55
***Neonatal characteristics***					
**Birthweight, grams, median (range)**	3190 (400–4380)	1400 (400–2900)	3285 (665–4380)	2	**<0.01**
**Gestational age at birth, days, median (range)**	272 (182–292)	241 (185–276)	273 (182–292)	2	**<0.01**
**Gender, male (%)**	98 (50.5)	11 (50)	87 (50.6)	0	0.82

General characteristics of the study population and the subgroups fetal growth restriction and controls. Data is presented as median and range or number (n) and percentage (%). Significant differences are in bold font. FGR, fetal growth restriction; BMI, body mass index in kilograms/square meter; IVF/ICSI, in vitro fertilization/intra-cytoplasmic sperm injection.

The estimated time to perform one ultrasound was about 40 minutes to a maximum of 60 minutes depending on the position of the fetus and quality of the ultrasound. Within this time we usually were able to perform the biometry measurements, Doppler measurements and the 3D-US sweeps for the brain fissure depth measurements. Measuring the brain fissure depths offline took about 15 minutes per ultrasound.

S1 Table shows the success rates, medians and ranges for the three brain fissure depths per GA. S2 Table shows the success rates, medians and ranges for the head biometry measurements.

### Longitudinal analyses

**Fig 2:**
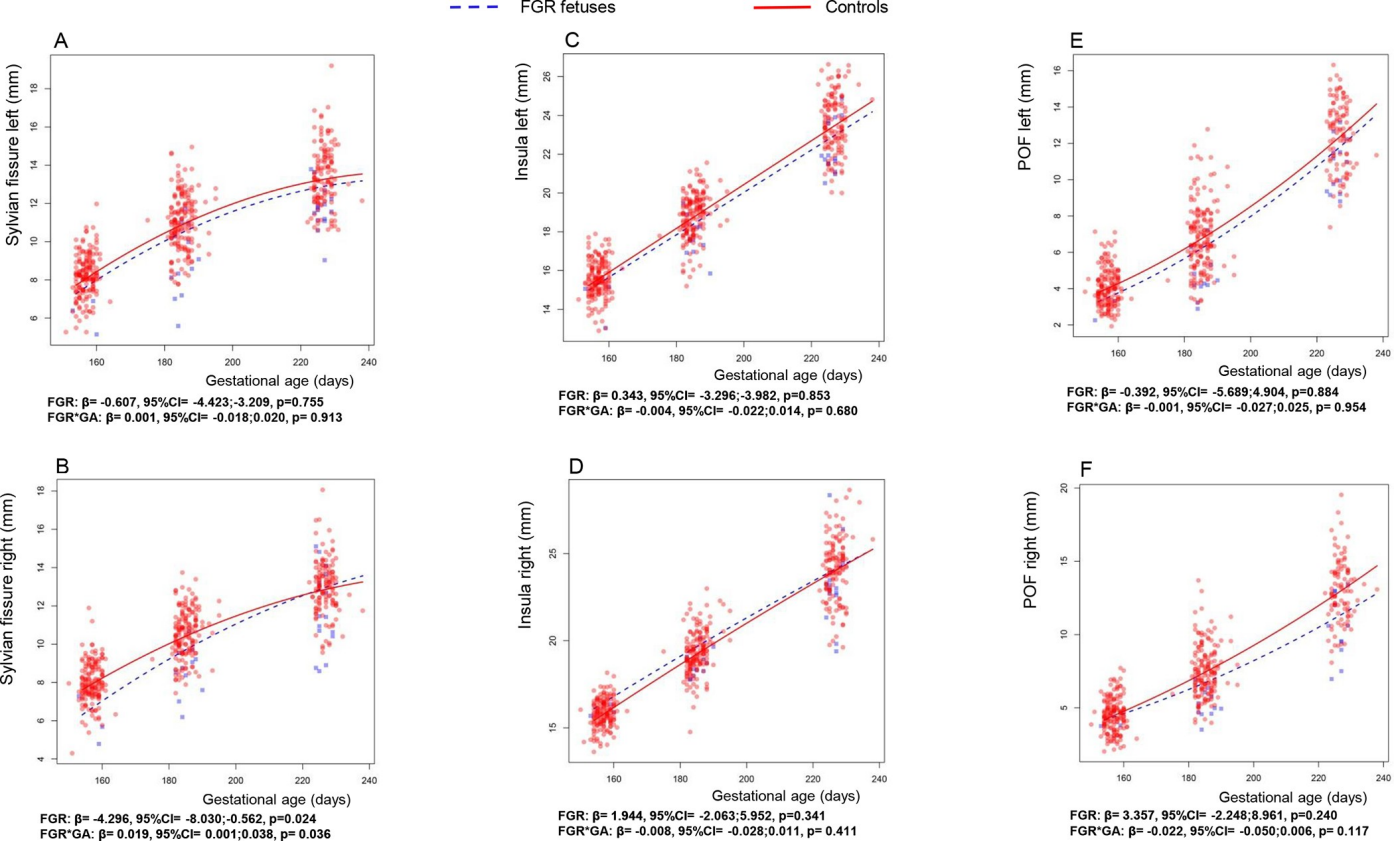
Trajectories of fetal brain fissure depths of FGR and controls. Data points and regression lines are depicted for the mean trajectories of the three brain fissures of FGR fetuses (blue) and controls (red) as a function of gestational age in days corresponding to the multivariate linear mixed models (model 2). Beta values FGR (growth) correspond to the mean difference between the FGR trajectories compared to controls. Beta values of FGR*GA (growth rate) correspond to the difference in mean slope per day gestational age of the FGR trajectory compared to controls. POF, Parieto-occipital fissure; FGR, fetal growth restriction; GA, gestational age; β, beta value, 95%CI, ninety-five percent confidence interval; p, p-value.

Table 2 shows the results of the crude and the fully adjusted linear mixed models. The growth trajectory of the right Sylvian fissure showed a significantly negative association with FGR fetuses compared to controls (ß = -4.31, 95%CI = -7.894;-0.720, p = 0.02) (Model 1). Adjustment for GA, HC, gender, educational level and parity showed comparable results (ß = -4.30, 95%CI = -8.03; -0.56, p = 0.024), while the growth rate in millimetres per day (FGR*GA) of the right Sylvian fissure was slightly increased in FGR compared to controls (ß = 0.02, 95%CI = 0.00; 0.04, p = 0.04) (Model 2). No significant associations were found between FGR fetuses and the growth trajectories of the Insula, POF and left Sylvian fissure. In the figures we show the mean trajectories of the left and right Sylvian fissure (Fig 2A and 2B), the left and right Insula (Fig 2C and 2D) and left and right POF (Fig 2E and 2F) based on our data points. In blue we depict the trajectories of FGR fetuses and in red the controls.

**Table 2:** Associations between FGR and longitudinal brain fissure depth measurements using linear mixed models.

			Model 1			Model 2			Model 3	
		β	95% CI	P	β	95% CI	P	β	95% CI	P
Sylvian Left	FGR	-2.788	-6.496; 0.919	0.140	-0.607	-4.423; 3.209	0.755	-0.544	-4.387; 3.299	0.781
	FGR * GA	0.004	-0.136; 0.023	0.627	0.001	-0.018; 0.020	0.913	0.001	-0.018; 0.019	0.948
	Sex male	0.468	-	**0.010**	0.151	-	0.325	0.113	-	0.473
	Education low	1.650	-	0.266	0.087	-	0.711	0.034	-	0.890
	Education middle	1.445	-	0.326	-0.104	-	0.525	-0.092	-	0.582
	Parity	-0.082	-	0.658	-0.168	-	0.271	-0.130	-	0.410
	CPR	0.322	-	0.097	-	-	-	-0.134	-	0.490
	HC	0.077	-	**<0.01**	0.070	-	**<0.01**	0.072	-	**<0.01**
Sylvian Right	FGR	-4.307	-7.894; -0.720	**0.019**	-4.296	-8.030; -0.562	**0.024**	-4.486	-8.182; -0.790	**0.017**
	FGR * GA	0.0128	-0.005; 0.030	0.151	0.019	0.001; 0.038	**0.036**	0.021	0.003; 0.039	**0.025**
	Sex male	0.368	-	**0.027**	0.131	-	0.352	0.118	-	0.398
	Education low	3.965	-	**<0.01**	0.147	-	0.497	0.184	-	0.399
	Education middle	3.959	-	**<0.01**	0.091	-	0.541	0.084	-	0.572
	Parity	0.172	-	0.297	0.100	-	0.477	0.027	-	0.849
	CPR	0.594	-	**<0.01**	-	-	-	0.157	-	0.383
	HC	0.066	-	**<0.01**	0.058	-	**<0.01**	0.056	-	**<0.01**
Insula Left	FGR	-0.685	-4.142; 2.772	0.697	0.343	-3.296; 3.982	0.853	0.380	-3.282; 4.042	0.838
	FGR * GA	-0.002	-0.019; 0.015	0.796	-0.004	-0.022; 0.014	0.680	-0.004	-0.022; 0.014	0.688
	Sex male	0.532	-	**<0.01**	0.402	-	**<0.01**	0.413	-	**<0.01**
	Education low	2.486	-	**0.042**	-0.134	-	0.474	-0.131	-	0.502
	Education middle	2.558	-	**0.035**	0.005	-	0.967	-0.007	-	0.960
	Parity	0.027	-	0.840	0.024	-	0.843	-0.019	-	0.877
	CPR	0.463	-	**<0.01**	-	-	-	0.155	-	0.394
	HC	0.042	-	**<0.01**	0.031	-	**<0.01**	0.030	-	**<0.01**
Insula Right	FGR	0.744	-3.051; 4.539	0.700	1.944	-2.063; 5.952	0.341	1.981	-2.060; 6.023	0.336
	FGR * GA	-0.007	-0.026; 0.011	0.444	-0.008	-0.028; 0.011	0.411	-0.008	-0.028; 0.012	0.415
	Sex male	0.330	-	**0.024**	0.143	-	0.309	0.148	-	0.305
	Education low	2.567	-	0.055	0.242	-	0.261	0.220	-	0.330
	Education middle	2.234	-	0.077	0.011	-	0.940	-0.022	-	0.888
	Parity	-0.259	-	0.081	-0.253	-	0.068	-0.225	-	0.122
	CPR	0.368	-	**0.046**	-	-	-	0.108	-	0.585
	HC	0.048	-	**<0.01**	0.052	-	**<0.01**	0.051	-	**<0.01**
POF Left	FGR	-2.602	-7.469; 2.264	0.294	-0.392	-5.689; 4.904	0.884	-0.342	-5.680; 5.000	0.900
	FGR * GA	0.004	-0.020; 0.028	0.744	-0.001	-0.027; 0.025	0.954	-0.001	-0.027; 0.025	0.946
	Sex male	0.226	-	0.261	0.015	-	0.940	-0.016	-	0.937
	Education low	2.285	-	0.172	-0.315	-	0.295	-0.358	-	0.259
	Education middle	2.711	-	0.102	0.075	-	0.714	0.071	-	0.739
	Parity	0.146	-	0.469	0.125	-	0.514	0.094	-	0.739
	CPR	0.743	-	**<0.01**	-	-	-	0.166	-	0.513
	HC	0.063	-	**<0.01**	0.052	-	**<0.01**	0.049	-	**<0.01**
POF Right	FGR	0.247	-5.023; 5.517	0.927	3.357	-2.248; 8.961	0.240	3.171	-2.469; 8.811	0.270
	FGR * GA	-0.012	-0.038; 0.013	0.346	-0.022	-0.050; 0.006	0.117	-0.021	-0.049; 0.007	0.139
	Sex male	0.327	-	0.125	0.138	-	0.493	-0.107	-	0.519
	Education low	5.045	-	**<0.01**	-0.336	-	0.272	-0.295	-	0.353
	Education middle	5.515	-	**<0.01**	0.155	-	0.464	0.177	-	0.413
	Parity	0.296	-	0.164	0.262	-	0.185	0.193	-	0.346
	CPR	0.840	-	**<0.01**	-	-	-	0.287	-	0.271
	HC	0.066	-	**<0.01**	0.044	-	**<0.01**	0.039	-	**<0.01**

Model 1 = Fissure = GA+ GA2 + covariate of interest

Model 2 = Multivariate: Fissure = GA + GA2 + Case + Case * GA + gender + education (low/middle) + parity + HC

Model 3 = Model 2 + CPR

Significant results are in bold. Model 1 represents the crude models investigating all covariates separately, model 2 is the multivariate model adjusted for educational level, parity, fetal gender and HC, model 3 is the multivariate model also adjusted for CPR. ß, beta value; 95%CI, ninety-five percent confidence interval; P, p-value; FGR, fetal growth restriction; GA, gestational age; POF, parieto-occipital fissure; HC, head circumference; CPR, cerebro-placental ratio.

Significantly positive associations were shown between HC and all brain fissures in the crude and fully adjusted model. Male gender was significantly positively associated with the Sylvian fissure depth and insula depth. In the multivariate model this association only remained significant in the trajectory of the left Insula.

CPR was positively associated with the Insula, POF and right Sylvian fissure depth. After adjustment for CPR in a multivariate model (Model 3) no significant association of CPR with the trajectories of all brain fissures was demonstrated in FGR fetuses.

## Discussion

### Main findings

In this study we show that brain fissure depth can be measured by ultrasound in FGR fetuses. These measurements might possibly serve as a noninvasive imaging marker to assess differences in fetal cortical folding between FGR fetuses and controls in the future. In FGR fetuses the trajectory of the right Sylvian fissure is significantly decreased in model 1 (adjusted for GA) and the fully adjusted model (Model 2). The growth rate of the right Sylvian fissure was slightly increased in FGR compared to controls in the fully adjusted model. No significant differences in trajectories of the left Sylvian, the insula and POF were found between FGR fetuses and controls. Male gender was significantly positive associated with trajectories of the Sylvian fissure and insula. HC was significantly positive associated with the trajectories of all brain fissure depths. In our study no associations were found between CPR and the trajectories of all brain fissures in FGR fetuses after adjustment.

The table of measurements shows that the range of Sylvian fissure measurements seems to be broader in the control group compared to the FGR group, specifically at 22 and 26 weeks of gestation. An explanation could be that the control group seems to be a more heterogeneous group. Table 1 also shows a bigger range of birth weight and GA at birth in the control group which supports this explanation.

### Strengths and limitations

Strengths of our study are the prospective cohort design and the use of 3D-US, which provides a precise method for brain fissure depth measurements by the reconstruction of orthogonal planes. 3D-US is an easy, cheap and accessible technique using standard axial ultrasound planes that can be performed serial in contrast to MRI. To limit interrater variability all measurements were performed by one trained observer (AWG). Multivariate models allow adjustment for possible confounders described in previous literature, i.e. HC and gender [22, 34]. By adjusting for HC we were able to differentiate between decreased brain fissure depth measurements due to the fetal growth restriction itself and smaller brain fissures as consequence of a smaller head size. The positive association of male gender and brain fissure depth measurements is in agreement with the physiology of brain development and previous literature [16, 22].

Conclusions of our study are limited to regional cortical folding, since we measured only three brain areas in our study. Because of the sample size, lack of postnatal neurodevelopmental follow-up, observational design of the study with potential threat of residual confounding, conclusions on causality cannot be drawn and the clinical implications have to be further investigated.

Unfortunately we did not define which side of the brain was the distal or proximal side per fetus. Differentiation between failed measurements of the distal and proximal side is therefore not possible in our study population. We hypothesize that the distal and proximal measurements are divided random between the groups and this will explain the small differences between the success rates of left and right per brain fissure depth measurement.

We assume that different position of the fetus might lead to a slight deformation of the skull and consequently result in a different fissure depth measurements. However, the many possible different positions of the fetal head make it impossible to correct for all those positions both in clinical practice and in research settings. Therefore no corrections are made for fetal position, not in the FGR group not in the control group.

The amount of cerebrospinal fluid (CSF) may influence the obtained brain fissure depth measurements. BPD and HC are standardized measurements and not adjusted for the amount of CSF in clinical practice. Unfortunately ultrasound does not allow us to correct for the amount of CSF. Our results show that the trajectory of the right Sylvian fissure is decreased in FGR fetuses, which means that the measurement from the internal border of the cortex to the inner border of the parietal bone, perpendicular to the midline, is smaller compared to the measurement in controls. If we take into account that CSF might be increased in FGR, it means that we have underestimated the effect and that in fact the “real” measurement of the Sylvian fissure is even more decreased.

In our study population there were only three FGR fetuses with CPR values lower than 1.0 at 32 weeks GA. Although this rather small numbers limits the conclusion that can be drawn, this finding suggests that the small regional differences in cortical folding can occur without finding prenatal associations between hemodynamic features of cerebro-placental redistribution. Potentially, a normal CPR does not guarantee proper cerebral perfusion.

Because there were only three FGR measurements at week 22 the estimated outcomes and the effect FGR at this age are not very precise. This low number of observations is why we only looked at a linear function for the difference between FGR and the controls. One might be worried that these three observations can have a large influence on the results. For that reason we have performed regression diagnostics. We concluded that while for a few of the outcomes the three FGR measurements in week 22 where indeed among the most influential observations, they never stuck out in comparison with the influence of the others nor was the amount of influence that they had high enough to be reason for concern.

The difference we find between FGR and controls is only seen in the trajectory of the right Sylvian fissure and not in the other fissures investigated in our study population. Whether we could interpret this finding as a delay or a disturbance remains unanswered. Larger studies and follow-up studies are necessary to investigate further whether other fissures are involved. Normal asymmetry in the formation of fissures between the right and left cerebral hemispheres however has been described in previous literature and confirms our findings **[11].** More research however has to be performed to show whether differences in the disturbance/delay in development between fissures can be explained by differences in timing of the development of the fissures.

Studying the effects of FGR is challenging as this field of research is still struggling to publish consistent definitions of growth restriction and babies’ small for gestation. In this study we classified FGR as fetuses with an AC or EFW below the 5^th^ percentile at the moment of inclusion. However, the majority of these FGR fetuses may not be pathologically growth restricted, but constitutionally small [35]. Therefore this classification may lead to false-positive cases. Although we already see small changes in our group of FGR fetuses, it is important in future to extend the study group with cases affected by placental insufficiency and fetal hypoxia to determine whether severe growth restricted fetuses are at risk for abnormal cortical folding. The cerebro-placental redistribution of fetal blood flow, characterized by abnormal Doppler indices in these FGR fetuses, affects many organs, including the central nervous system and may cause impaired neurodevelopment [35]. Previous literature describes even worse perinatal outcomes for FGR fetuses with circulatory redistribution than FGR fetuses without circulatory redistribution [8, 35, 36]. Morales-Rosello et al. describes a third group of fetuses at risk only determined by abnormal Doppler indices of the MCA and UA called appropriate-for-gestational-age (AGA) fetuses that are failing to reach their growth potential (FRGP). Fetal circulatory redistribution or CPR may be a good marker for severity of placental insufficiency and the severity of the risk of neurodevelopmental impairment in growth-restricted fetuses [35, 36]. However, this has to be investigated in future research.

### Interpretation

This study presents longitudinal data of fetal brain fissure depth measurements using 3D-US and describes the accelerated growth rate of the right Sylvian fissure in FGR fetuses. The decreased growth trajectory of the right Sylvian fissure implies a delay in cortical folding in FGR fetuses. However, previous prenatal and postnatal imaging studies do not show the same results. The regional developmental delay of the right sylvian fissure is in contrast with Egana et al. who described significantly deeper left and right insular and left cingulate depths, decreased brain volume, a thinner insular cortex and smaller insula volume and worse neurobehavioral outcome in term small for gestational age neonates [1, 2]. A reduction in cortical grey matter and altered cortical maturation was demonstrated in fetuses and preterm infants with IUGR in previous studies [4, 37]. The diversity of the reported findings may be explained by the wide variety of measures and methods used to evaluate fetal brain development. The wide variety and limited standardization of methods as well as the differences and heterogeneity of study populations evaluating cortical folding may explain the observed discrepancies in reported findings of normal development of cortical folding [38]. One assumes that this also accounts for abnormal brain development.

## Conclusion

With this explorative study we try to push the boundaries towards a more subtle look at the developing fetus, especially of the brain development in FGR. Not only growth itself, but also brain development seems to be different in this fetuses. We show that subtle measurements in the fetal brain can be performed reliable and seem to be different between FGR and controls already in the second trimester of pregnancy. This should be considered as the first step towards screening FGR fetuses using these 3D ultrasound brain measurements and to perform future research on what will be the consequence of these differences. Improvement of fetal management techniques contributes to a higher survival rate of FGR fetuses with higher rates of childhood morbidity [36]. One of these morbidities associated with FGR is abnormal neurodevelopmental outcome [1, 36]. We argue that identification of fetuses at risk for neurodevelopmental impairment prenatally is essential to improve pre- and postnatal management and define strategies to improve neurodevelopmental outcome. However, replication of our study is essential to investigate whether our method can be used for this purpose in the future. Our results imply the presence of prenatal developmental changes of the brain in in FGR fetuses. However, whether these small regional changes are of clinical relevance for postnatal neurodevelopmental outcome needs to be elucidated. For this purpose studies with a larger sample size to increase power and postnatal follow-up is necessary.

## Supporting information

S1 Table Success rates and the medians of brain fissure depth measurements. Success rates of fissure measurements, the median in millimeters (mm), per gestational age in weeks. GA, gestational age; wks, weeks; n, number; %, percentage; mm, millimeters; POF, parieto-occipital fissure.
(DOCX)

S2 Table Success rates and the medians of the head biometry measurements. Success rates of head circumference and biparietal diameter, the median in millimeters (mm), per gestational age in weeks. GA, gestational age; wks, weeks; n, number; %, percentage; mm, millimeters; HC, head circumference; BPD, biparietal diameter.
(DOCX)

S1 File Minimal data set. (XLSX)
