# Supplementary material for: Three-dimensional ultrasound imaging of fetal brain fissures in the growth restricted fetus
Source: PLoS One. 2019 May 23;14(5):e0217538. doi: 10.1371/journal.pone.0217538 (PMC6532926; doi:10.1371/journal.pone.0217538)
Supplement: S1 Table — Success rates of fissure measurements, the median in millimeters (mm), per gestational age in weeks. GA, gestational age; wks, weeks; n, number; %, percentage; mm, millimeters; POF, parieto-occipital fissure. (DOCX) [file pone.0217538.s001.docx]

**S1 Table. Success rates and the medians of brain fissure depth measurements.**

| **GA (wks)** | **Brain fissures** | **Brain fissure measurements** | | |  |  | |  |  | |  | | |  | |  |
| --- | --- | --- | --- | --- | --- | --- | --- | --- | --- | --- | --- | --- | --- | --- | --- | --- |
|  |  | **Total** | | | | **FGR** | | | | **Control** | | | | |  |  |
|  |  | n (%) | median (mm) | range | | n (%) | median (mm) | range | | n (%) | | median (mm) | range | |  |  |
| 22 | Left Sylvian | 167 (95) | 8.21 | 5.15 - 11.97 | | 3 (100) | 6.40 | 5.15 - 6.89 | | 164 (95) | | 8.24 | 5.27 - 11.97 | |  |  |
|  | Right Sylvian | 162 (93) | 7.88 | 4.30 - 11.89 | | 3 (100) | 5.68 | 4.79 - 7.28 | | 159 (92) | | 7.89 | 4.30 - 11.89 | |  |  |
|  | Left insula | 167 (95) | 15.48 | 12.90 - 17.89 | | 3 (100) | 15.06 | 13.04 - 15.16 | | 164 (95) | | 15.50 | 12.90 - 17.89 | |  |  |
|  | Right insula | 162 (93) | 15.84 | 13.62 - 18.16 | | 3 (100) | 15.91 | 15.69 - 16.36 | | 159 (92) | | 15.84 | 13.62 - 18.16 | |  |  |
|  | Left POF | 168 (96) | 3.93 | 1.93 - 7.14 | | 2 (67) | 2.79 | 2.26 - 3.32 | | 166 (97) | | 4.00 | 1.93 - 7.14 | |  |  |
|  | Right POF | 167 (95) | 4.42 | 2.03 - 7.72 | | 2 (67) | 3.79 | 3.76 - 3.81 | | 165 (96) | | 4.45 | 2.03 - 7.72 | |  |  |
| 26 | Left Sylvian | 178 (97) | 10.96 | 5.59 - 14.95 | | 12 (100) | 8.83 | 5.59 - 11.74 | | 166 (97) | | 11.03 | 7.78 - 14.95 | |  |  |
|  | Right Sylvian | 174 (95) | 10.48 | 6.19 - 13.74 | | 11 (92) | 8.76 | 6.19 - 10.66 | | 163 (95) | | 10.60 | 7.44 - 13.74 | |  |  |
|  | Left insula | 178 (97) | 18.92 | 15.20 - 21.56 | | 12 (100) | 17.61 | 15.85 - 19.47 | | 166 (97) | | 18.95 | 15.20 - 21.56 | |  |  |
|  | Right insula | 174 (95) | 19.24 | 14.76 - 22.99 | | 11 (92) | 18.37 | 17.75 - 20.17 | | 163 (95) | | 19.30 | 14.76 - 22.99 | |  |  |
|  | Left POF | 167 (91) | 6.44 | 2.89 - 12.78 | | 11 (92) | 4.28 | 2.89 - 7.24 | | 165 (97) | | 6.50 | 3.23 - 12.78 | |  |  |
|  | Right POF | 173 (95) | 7.22 | 3.52 - 13.70 | | 11 (92) | 4.99 | 3.52 - 6.93 | | 162 (95) | | 7.39 | 3.98 - 13.70 | |  |  |
| 32 | Left Sylvian | 145 (82) | 13.13 | 9.03 - 19.20 | | 15 (94) | 11.65 | 9.03 - 13.79 | | 130 (81) | | 13.25 | 10.61 - 19.20 | |  |  |
|  | Right Sylvian | 146 (83) | 13.01 | 8.59 - 18.06 | | 15 (94) | 11.42 | 8.59 - 15.11 | | 131 (81) | | 13.06 | 9.56 - 18.06 | |  |  |
|  | Left insula | 145 (82) | 23.39 | 20.00 - 26.63 | | 15 (94) | 21.78 | 20.51 - 24.87 | | 130 (81) | | 23.49 | 20.00 - 26.63 | |  |  |
|  | Right insula | 146 (83) | 24.07 | 19.39 - 28.65 | | 15 (94) | 23.01 | 19.39 - 28.35 | | 131 (81) | | 24.19 | 19.62 - 28.65 | |  |  |
|  | Left POF | 117 (66) | 12.41 | 7.38 - 16.32 | | 13 (81) | 10.99 | 8.81 - 13.16 | | 104 (65) | | 12.75 | 7.38 - 16.32 | |  |  |
|  | Right POF | 110 (62) | 12.85 | 6.97 - 19.54 | | 11 (69) | 10.60 | 6.97 - 13.42 | | 99 (62) | | 12.96 | 8.09 - 19.54 | |  |  |

*Success rates of fissure* *measurements, the median in millimeters (mm), per gestational age in weeks. GA, gestational age; wks, weeks; n, number; %, percentage; mm, millimeters; POF, parieto-occipital fissure.*
