# Supplementary material for: Three-dimensional ultrasound imaging of fetal brain fissures in the growth restricted fetus
Source: PLoS One. 2019 May 23;14(5):e0217538. doi: 10.1371/journal.pone.0217538 (PMC6532926; doi:10.1371/journal.pone.0217538)
Supplement: S2 Table — Success rates of head circumference and biparietal diameter, the median in millimeters (mm), per gestational age in weeks. GA, gestational age; wks, weeks; n, number; %, percentage; mm, millimeters; HC, head circumference; BPD, biparietal diameter. (DOCX) [file pone.0217538.s002.docx]

**S2 Table . Success rates and the medians of the head biometry measurements.**

| **GA (wks)** |  |  | | |  |  | |  |  |  | |
| --- | --- | --- | --- | --- | --- | --- | --- | --- | --- | --- | --- |
|  |  | **Total** | | | | **FGR** | | | **Control** | | |
|  |  | n (%) | median (mm) | range | | n (%) | median (mm) | range | n (%) | median (mm) | range |
| 22 | HC | 175 (100) | 200.5 | 181.5 - 220.5 | | 3 (100) | 183.1 | 181.5 - 188.1 | 172 (100) | 200.7 | 183.9 - 220.5 |
|  | BPD | 175 (100) | 55.6 | 48.6 - 63.5 | | 3 (100) | 50.9 | 50.9 - 56.1 | 172 (100) | 55.6 | 48.6 - 63.5 |
| 26 | HC | 183 (100) | 246.5 | 200 - 273.1 | | 12 (100) | 223.8 | 200 - 243.4 | 171 (100) | 246.7 | 229.9 - 273.1 |
|  | BPD | 183 (100) | 68.9 | 55.9 - 79.2 | | 12 (100) | 62.1 | 55.9 - 69.7 | 171 (100) | 69 | 62.7 - 79.2 |
| 32 | HC | 177 (100) | 296.8 | 258.6 - 324.3 | | 16 (100) | 280.7 | 258.6 - 295.3 | 161 (100) | 298.4 | 274.3 - 324.3 |
|  | BPD | 177 (100) | 84.9 | 72 - 94.4 | | 16 (100) | 80.2 | 72 - 82.6 | 161 (100) | 85.3 | 77.9 - 94.4 |

*Success rates of head circumference and biparietal diameter, the median in millimeters (mm), per gestational age in weeks. GA, gestational age; wks, weeks; n, number; %, percentage; mm, millimeters; HC, head circumference; BPD, biparietal diameter.*
